# After-Hours Use of the Electronic Health Record Among Medical and Surgical Specialists After Implementation of a System-Wide Integrated Clinical Information System in Alberta, Canada: Longitudinal Descriptive Study

**DOI:** 10.2196/76872

**Published:** 2026-04-15

**Authors:** Robert P Pauly, Tania Stafinski, Helia Koosha, Melita Avdagovska, Maliheh Hadizadeh, David Bigam, Timothy Graham, Craig Kuziemsky, Narmin Kassam, Devidas Menon

**Affiliations:** 1 Department of Medicine, University of Alberta, 11-107 Clinical Sciences Building, 8440 112 Street NW, Edmonton, AB, T6B 2B7, Canada, 1 780-492-3318; 2 School of Public Health, University of Alberta, Edmonton, AB, Canada; 3 Faculty of Engineering, University of Alberta, Edmonton, AB, Canada; 4 Department of Surgery, University of Alberta, Edmonton, AB, Canada; 5 Department of Emergency Medicine, University of Alberta, Edmonton, AB, Canada; 6 Office of Research Services, MacEwan University, Edmonton, AB, Canada; 7 School of Business, MacEwan University, Edmonton, AB, Canada

**Keywords:** electronic health record, EHR, after hours, pajama time, time outside scheduled hours, TOSH, efficiency, workload, specialists, clinicians, longitudinal, Epic, Canada, Edmonton, quaternary, hospital, medical, surgical, informatics, burnout, well-being, workflow

## Abstract

**Background:**

Studies suggest that the introduction of electronic health records (EHRs) has decreased the efficiency of clinical practice and increased clinician workload for US-based physicians. Most studies involve clinicians in primary care settings. Less is known about other health care settings, subspecialist clinicians, or whether markers of efficiency and workload change over time.

**Objective:**

This study aimed to describe 2 common metrics of after-hours use of the EHR (pajama time and time outside scheduled hours [TOSH]) among diverse specialists and track these parameters longitudinally in a Canadian setting.

**Methods:**

In this longitudinal descriptive study, medical and surgical specialists were observed starting from the introduction of a system-wide EHR in 2019 to 2022 at a large quaternary teaching hospital in Edmonton, Alberta. Pajama time and TOSH were extracted from the EHR on an Epic system platform and monitored over time. Clinicians were stratified according to clinical group (medical and surgical) and workload (clinical full-time equivalent).

**Results:**

A total of 71 medical and surgical specialists participated in this study, spending approximately 24 to 40 minutes per day on pajama time and 32 to 55 minutes per day on TOSH depending on clinician grouping. Both metrics increased over the observation period, as reflected in the longitudinal plots and the higher values observed at the end vs the beginning of follow-up.

**Conclusions:**

After-hours EHR use in this Canadian cohort of medical and surgical specialists was similar to what is reported in the US literature, although the drivers may be different. Perhaps surprisingly, these markers increased over time despite presumed improved familiarity with the EHR. The extent to which this affects clinician well-being and work-life integration cannot be determined from these results, although there may be cause for concern.

## Introduction

The purported benefits of implementing electronic health records (EHRs) in the past decade were to decrease costs, more effectively manage health information, increase guideline adherence, and reduce medical errors [1,2]. However, delivering on these promises has come at the expense of other aspects of care, such as increased clinician workload and decreased efficiency [1,3,4]. The impact of EHRs on clinician well-being can be viewed through the lens of the job demands–resources (JD-R) framework, where well-being is the integration of job demands and available resources [5]. EHRs are an important resource and simultaneously represent a cognitive burden with inbox management, order entry, and documentation; a temporal burden with EHR work encroaching on traditional nonwork time; and a boundary-blurring strain on work-life balance with few constraints on system accessibility and system engagement expectation. Studies suggest that clinicians spend considerable time on the EHR, particularly after typical work hours, such as on evenings and weekends [6,7]. This after-hours engagement with the system is also called work outside work [8] and has even led to the coining of a new term, *pajama time*, to describe the time at home in the evening when clinicians re-engage with the EHR to complete tasks that would otherwise have been done at the office during regular working hours. After-hours work such as this may impact clinician well-being, an area that is receiving more attention as the physician workforce is facing increasing pressures from within health systems and the public, with significant rates of burnout reported in Canada, the United States, and elsewhere [9-12]. In fact, there is an increasing body of evidence that links the excessive burden of EHR use directly with burnout and other measures of clinician dissatisfaction [13-16]. The specific aspects of the EHR that drive this dissatisfaction are less clear, although after-hours engagement with the electronic system is likely a key contributor [17-20]. An important limitation of the existing literature investigating EHR use is that most studies have been conducted in the United States, where documentation is influenced in large part by insurance claim requirements, billing, quality reporting, and litigation risks. These drivers of documentation (and, hence, use of the EHR) vary from country to country and could easily result in systematic differences in clinician documentation behavior in different health care systems. This raises the question of whether the widely reported results of EHR use in general and use outside of regular working hours in particular are generalizable to other health systems.

In 2019, Alberta Health Services began system-wide implementation of a comprehensive EHR in Alberta, Canada. This provided a unique opportunity to track clinician EHR use longitudinally in a Canadian setting. This study aimed to describe 2 common metrics of after-hours use, pajama time and work time outside scheduled hours (TOSH), and track trends over time among medical and surgical specialists in a large quaternary care setting after the implementation of the EHR. Ours is a descriptive study summarizing differences and temporal patterns in aggregated monthly measures rather than aiming to test prespecified causal hypotheses.

## Methods

### Study Design, Setting, and Participants

This was a longitudinal descriptive study conducted at the University of Alberta Hospital in Edmonton, Alberta. We collected physician EHR use data between November 1, 2019 (system rollout), and July 30, 2022. A more detailed study protocol was previously published [21]. A purposive sampling method was used to recruit clinicians from the Department of Medicine and the Department of Surgery inclusive of all medical and surgical subspecialists. Specifically, the study aims and methodology were advertised and promoted during departmental rounds, and participants were tracked using REDCap (Research Electronic Data Capture; Vanderbilt University). The study population consisted of clinicians who had an ambulatory care practice and at least 7 months of experience with the EHR. Clinicians were asked to identify their department or division, EHR username (to link individuals to their EHR data), gender, and self-reported clinical full-time equivalent (FTE) percentage. The latter was used as a marker of overall clinical workload given that many clinicians also have substantial research, teaching, and administrative portfolios. No demographic or other personal information was obtained other than EHR use data (see below). Participants were grouped into medical specialists with a clinical FTE of 0.5 or less, medical specialists with a clinical FTE of more than 0.5, and surgical specialists with a clinical FTE of more than 0.5 (no surgeon reported a clinical FTE≤0.5).

### Ethical Considerations

The study protocol was approved by the University of Alberta Hospital Research Ethics Board (case number Pro00119194), and individual participants provided written consent allowing their anonymized EHR use data to be transferred from Alberta Health Services (the legal custodian of the EHR) to the study team for analysis. Participants were not compensated for taking part.

### Data Source and Metrics

Connect Care is the provincial EHR customized on an Epic platform (Epic Systems). Data were obtained from Signal, an analytics tool developed by Epic Systems using EHR user action log data and integrated into the EHR platform. Data related to outpatient visits were extracted in aggregate 1–calendar month reporting periods.

The two key metrics used to describe clinician engagement with the EHR outside typical ambulatory clinical working hours were (1) pajama time, defined as the average number of minutes that a clinician spent in charting activities on weekdays outside the hours of 7 AM to 5:30 PM or outside scheduled hours on weekends or nonscheduled holidays; and (2) TOSH, defined as the average number of minutes that a provider spent on the system outside of scheduled hours, where scheduled hours represented scheduled clinic appointments with a 30-minute time buffer before and after those scheduled clinic appointments. The calculation of these metrics is outlined in Table S1 in Multimedia Appendix 1. TOSH was captured for the duration of the study, whereas pajama time was captured from October 2020 to study termination in July 2022 as this metric was not available within the system for the first year after rollout (see Figure S1 in Multimedia Appendix 1 for a graphic representation of the observation periods).

System-imposed restrictions (ie, nonmodifiable Signal defaults) limited availability to only those data for which participants registered seeing at least 1 ambulatory care patient during the reporting period. There was an additional system-imposed restriction for one specific metric contributing to the total time that a participant spent engaged with the EHR: time in the in-basket per appointment. A participant needed to have 5 or more appointments per week within the reporting period to trigger the collection of this variable.

### Statistical Analysis

Data were summarized using SAS (version 9.4; SAS Institute) and Tableau (version 2021.4.3; Tableau Software LLC) as month-level group-weighted means for each metric to describe typical monthly group levels (ie, aggregated summaries rather than clinician-level longitudinal effects). For example, the monthly weighted mean of pajama time was calculated by summing the pajama time minutes of all participating clinicians in a given month and dividing it by the sum of the number of days that all clinicians spent on the system during that month. Because consecutive months are serially related in a longitudinal time series, we did not treat month-level summaries as independent observations for primary inference. Accordingly, the results are presented descriptively. Longitudinal figures display a 4-month simple moving average for visualization only. As a sensitivity analysis, we compared the distribution of month-level group summaries between groups using the Mann-Whitney *U* test; these results are reported as supportive only and should be interpreted cautiously given the independence assumption. Missing data were identified for pajama time for April 2021, July 2021, and September 2021 and for TOSH for April 2021; these months were excluded using listwise deletion.

## Results

A total of 71 participants from 19 medical and surgical specialties were enrolled in this study, with 27 (38.0%) being medical specialists with clinical FTE of 0.5 or less, 26 (36.6%) being medical specialists with clinical FTE of more than 0.5, and 18 (25.4%) being surgical specialists with clinical FTE of more than 0.5 (no surgeon reported a clinical FTE≤0.5). In total, 39.4% (28/71) of all specialists were women (details on specialist participants can be found in Table S2 in Multimedia Appendix 1).

**Figure 1.**
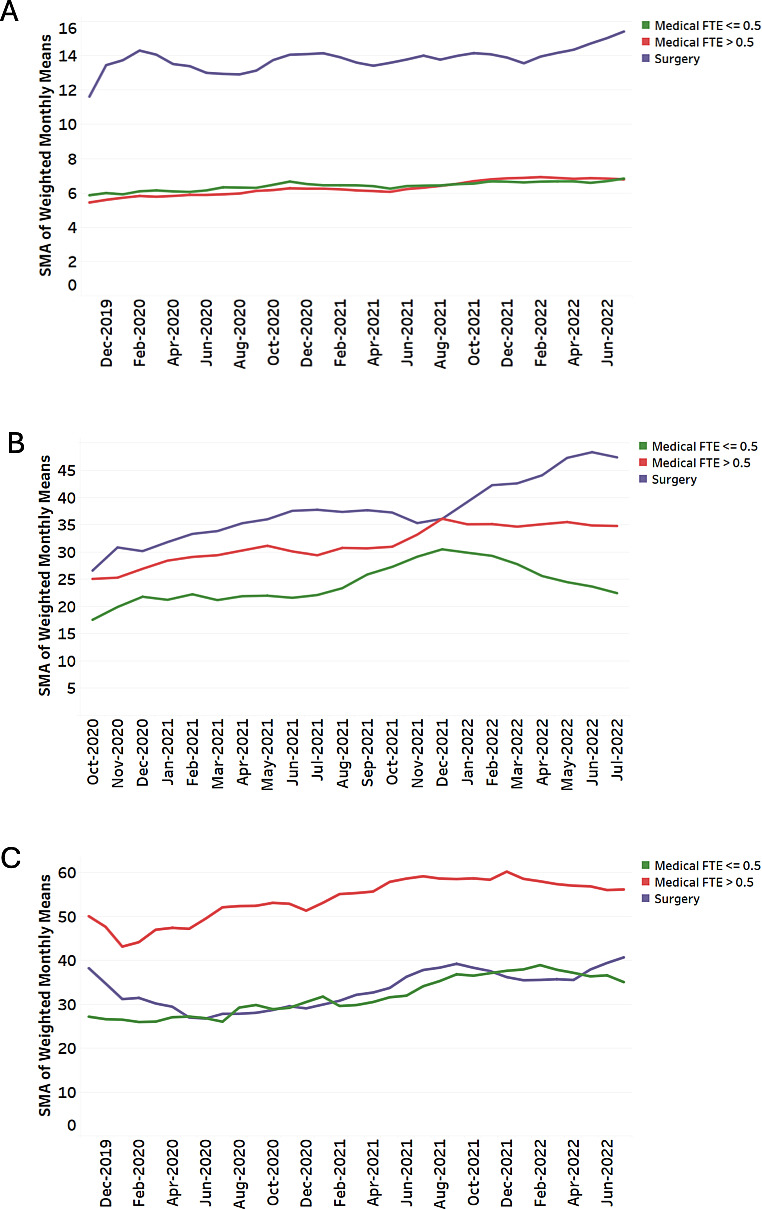
Longitudinal trends in outcome measures for (A) appointments per day, (B) pajama time, and (C) time outside scheduled hours. The green line corresponds to the medical group with clinical full-time equivalent (FTE) of 0.5 or less, the red line corresponds to the medical group with clinical FTE of more than 0.5, and the purple line corresponds to the surgical group. Graphs are shown as simple moving averages (SMAs).

The average number of appointments per day, pajama time, and TOSH is summarized in Table 1. Surgeons had a higher number of appointments per scheduled ambulatory day than medical specialists, and after-hours EHR time ranged from approximately 24 to nearly 40 minutes per scheduled day for pajama time and 32 to 55 minutes per scheduled day for TOSH depending on clinician group. Among medical specialists, clinicians with clinical FTE of 0.5 or less had lower after-hours EHR time than clinicians with clinical FTE of more than 0.5 for both pajama time and TOSH. Over the observation period, appointments per day, pajama time, and TOSH showed an overall upward pattern in all clinician groups (Table 2 and Figure 1). To provide an interpretable descriptive summary of change over time, Table 3 compares the average of the first 3 months vs the last 3 months of available data for pajama time and TOSH. For example, surgeons averaged 30.1 minutes of pajama time per scheduled day in the first 3 months and 47.3 minutes per scheduled day in the last 3 months of observation.

**Table 1.** Weighted monthly means of the reported metrics for medical and surgical groups.

Metric	All specialties (95% CI^a^)	Medical specialties (95% CI)			All surgical specialties^b^ (95% CI)
		All medical specialties	Medical specialties with FTE^c^ ≤0.5	Medical specialties with FTE >0.5	
Number of appointments per day	8.94 (8.20-9.67)	6.42 (6.31-6.52)	6.47 (6.33-6.61)	6.36 (6.20-6.52)	13.98 (13.50-14.47)
Pajama time (minutes)	32.10 (29.82-34.39)	28.21 (26.17-30.25)	24.05 (21.77-26.34)	32.37 (30.20-34.53)	39.89 (36.31-43.47)
Time outside scheduled hours (minutes)	42.27 (37.88-42.67)	43.44 (40.27-46.61)	32.33 (30.26-34.41)	54.54 (52.28-56.80)	33.94 (31.69-36.19)

a 95% CIs were calculated from the distribution of the month-level weighted means across the observation period for each group. These CIs reflect between-month variability of aggregated summaries.

b All surgeons reported a clinical full-time equivalent of more than 0.5.

c FTE: full-time equivalent.

**Table 2.** Trend analysis of the reported metrics over time for the medical and surgical groups.

Metric	Medical specialties with FTE^a^ ≤0.5		Medical specialties with FTE >0.5		Surgical specialties	
	Slope	Trend^b^	Slope	Trend	Slope	Trend
Appointments per day	0.0008	Increasing	0.0014	Increasing	0.0015	Increasing
Pajama time^c^	0.0118	Increasing	0.0161	Increasing	0.0275	Increasing
Time outside scheduled hours^d^	0.0140	Increasing	0.0137	Increasing	0.0098	Increasing

a FTE: full-time equivalent.

b Direction is based on the sign of the slope from a linear fit to the month-level series displayed for visualization (Figure 1). These trend summaries are descriptive and are not intended as confirmatory inference.

c Trend spanning October 2020 to July 2022.

d Trend spanning November 2019 to July 2022.

**Table 3.** Changes in pajama time and time outside scheduled hours (TOSH) over the observation period.

Specialist group	Pajama time (minutes per scheduled day), mean		TOSH (minutes per scheduled day), mean	
	First 3 months^a^	Last 3 months^b^	First 3 months^c^	Last 3 months^b^
Medical specialties with FTE^d^ ≤0.5	21.8	22.6	26.5	34.8
Medical specialties with FTE >0.5	26.9	34.8	43.1	56.4
Surgical specialties	30.1	47.3	31.2	43.6

a October 2020 to December 2020 inclusive.

b May 2022 to July 2022 inclusive.

c November 2019 to January 2020 inclusive.

d FTE: full-time equivalent.

## Discussion

To our knowledge, this is the only study to track physician after-hours EHR use in ambulatory care across multiple medical and surgical specialties from the time of EHR implementation for almost 3 years in a Canadian setting. Adoption of EHRs in Canada has accelerated in the past decade, but there is little to no quality publicly available data to benchmark clinician use; this study fills that void. Clinician workload, as measured using the number of appointments per clinic day, increased over time, accompanied by concurrent trends in increased after-hours engagement with the EHR regardless of clinician group or job description. The average amount of after-hours time that clinicians engaged with the EHR equated to approximately 11 to 19 hours per month for pajama time and approximately 15 to 25 hours per month for TOSH depending on specialty group.

This represents significant infringement on time that might reasonably be considered outside routine clinical service. Sinsky et al [6] reported after-hours EHR use at 53 minutes per day measured prospectively through clinician self-reporting for a diverse group of primary care and internal medicine specialists, although approximately a quarter of the evenings and nights under observation were skewed by clinicians also being on call (when one might anticipate accessing the EHR for patient care as part of expected duties). This compares to 1.4 hours per day per 1.0 clinical FTE reported by Arndt et al [7], 58 minutes per day per 1.0 clinical FTE reported by Attipoe et al [22], or 48 minutes per workday reported in the latter’s subsequent study [23]. These US-based studies are limited by their cross-sectional design. Our study found that both pajama time and TOSH increased over nearly 2 and 3 years since EHR implementation, respectively, which may be interpreted as a worrisome sign. Specifically, familiarity with the EHR presumably increased over time but coincided with increased after-hours engagement with the system rather than a decrease, as might have been reasonably expected.

However, the observation that after-hours clinician work in this study was within the general range observed in US studies indicates that Canadian clinicians are similar to their US colleagues, at least as far as their after-hours EHR use is concerned. This suggests that country differences in EHR engagement do not have as much of a net effect on clinician time as might be predicted (ie, specific documentation requirements in the United States appear to be balanced by other EHR activities in Canada, although this is speculative). Indeed, a recent multinational comparison of EHR use with the Epic Systems global ambulatory care customer base suggests that country-specific contextual differences play a significant role in EHR use [24].

There is no agreed-upon gold standard metric for after-hours EHR use [8]. Pajama time is tied to clock-based time and may be a reasonable marker of after-hours system engagement for clinicians with predictable schedules involving daytime ambulatory practice. This metric is not well suited for clinicians with varied inpatient and outpatient practices or shift work. TOSH, on the other hand, is tied to clinicians’ ambulatory care appointment schedules. At first glance, this may appear to solve the disadvantages of pajama time, but it is not well suited for clinicians with fragmented ambulatory clinic schedules or those whose clinical duties predominantly involve inpatient care [25]. Thus, comparisons of after-hours work between studies should be interpreted with caution. First, there is variation in how after-hours work is defined and assessed. Some studies cite clinician self-reported EHR time [6]. Others use externally unvalidated proprietary vendor predefined metrics (as is the case in this study) that variably use clock time–based metrics vs schedule-based metrics [24,26]; a combination of mouse clicks, keystrokes, and mouse miles (pixels) traveled to assess EHR activity [27]; or event logging data [22,23], which is believed to capture activity with the highest degree of precision but is expensive to perform and requires significant analytic power and expertise. Each of these approaches has its limitations [8]. Second, for studies capturing vendor-defined use metrics, there is no consistency in defining these metrics across platforms, and comparisons of EHR use from studies of health systems using different vendors remain unvalidated [25]. This is particularly relevant in this study: while pajama time and TOSH are well-recognized concepts, in our study, they are Epic-generated defaults, and the precise methods to calculate these may differ from those used by other vendors. Third, much of the literature on after-hours EHR use focuses on primary adult and pediatric care, with much less literature centered on specialist care. There is some evidence that surgeons, for example, have different use patterns from those of either primary care providers or medical specialists [26], a finding that this study corroborates. Fourth, there is no standardized method to account for physicians’ clinical workload and the bias that this may introduce into after-hours EHR use metrics. Some studies have normalized reporting to clinical FTE [7,19], whereas in most cases, normalization remains unreported or ambiguous [6,22-24,26,27]. Others have adjusted for relative value units, a metric used in the US health care system as a marker of volume and type of clinical service delivered [22]. Even comparing this study to the existing literature is imprecise and fraught due to different reporting methodologies. Finally, for those studies using event or access log metadata, there is no agreed-upon classification of EHR events that cumulatively defines activities that clinicians perform within the system.

Notwithstanding these sources of inherent bias encompassing the entire body of literature, this study has a number of important strengths. First, this is one of only a few reports from a non-US health system; therefore, it provides an opportunity to benchmark after-hours EHR use in a contemporary Canadian setting. Because this study includes a wide range of medical and surgical specialists, it also adds to only a small number of other studies investigating EHR use outside of primary care. Finally, this was a longitudinal study tracking changes in after-hours EHR use from EHR implementation for nearly 3 years, so we were able to report on changes over time. To our knowledge, all other studies save 1 [28] are cross-sectional. Interestingly, this sole other longitudinal study involved primary care physicians in the United States and also demonstrated increased after-hours EHR workload between 2019 and 2023.

Nonetheless, this study has several limitations. First, it is descriptive in nature and, thus, is agnostic to the impact of clinicians’ after-hours work on well-being, burnout, or work-life integration. Linking EHR use metrics to measures of professional satisfaction is a future research priority. Second, this study is likely to underestimate true after-hours EHR work because of underreporting of time due to EHR system-imposed restrictions on data capture. For example, a participant needed to have 5 or more appointments per week to trigger the collection of in-basket metrics (which, presumably, is expected to make up a meaningful proportion of the after-hours time that clinicians spend on the EHR during pajama time or TOSH)—thus raising concerns that pajama time and TOSH may be significantly underestimated for those clinicians with low ambulatory clinical volume, such as surgeons with irregular schedules or internists with high inpatient care service but little outpatient demands. Third, this study did not include any information on clinician schedules or patient complexity so that even comparisons between specialists are limited by lack of normalization to clinical activity. Fourth, the rollout of the EHR in Alberta (and, hence, this study) coincided with the COVID-19 pandemic, when routine ambulatory practice temporarily and intermittently transitioned to virtual care, where use of an EHR might well have shifted practice patterns, including after-hours EHR use. Fifth, data for pajama time were not available in the EHR for the first 12 months of observation, so this metric could not be reported from the time of system rollout in the same way as such baseline data were only available for TOSH. This may limit the interpretation of trends over time (Table 2), and it is not known whether this dampens or exaggerates the effect that we report. Sixth, because participants were recruited using purposive sampling, there is inherent self-selection bias in terms of clinicians opting to participate, potentially affecting generalizability to physicians at large. Generalizability may also be limited as study participants were derived from a single quaternary hospital setting among clinicians with an ambulatory practice, which may not represent after-hours use of the EHR among clinicians without an ambulatory practice or in community hospital settings. Finally, while this study is larger than most in this research space, a larger participant pool would have enabled more robust analysis of relevant strata of clinician ages, genders, and areas of specialization.

**Figure 2.**
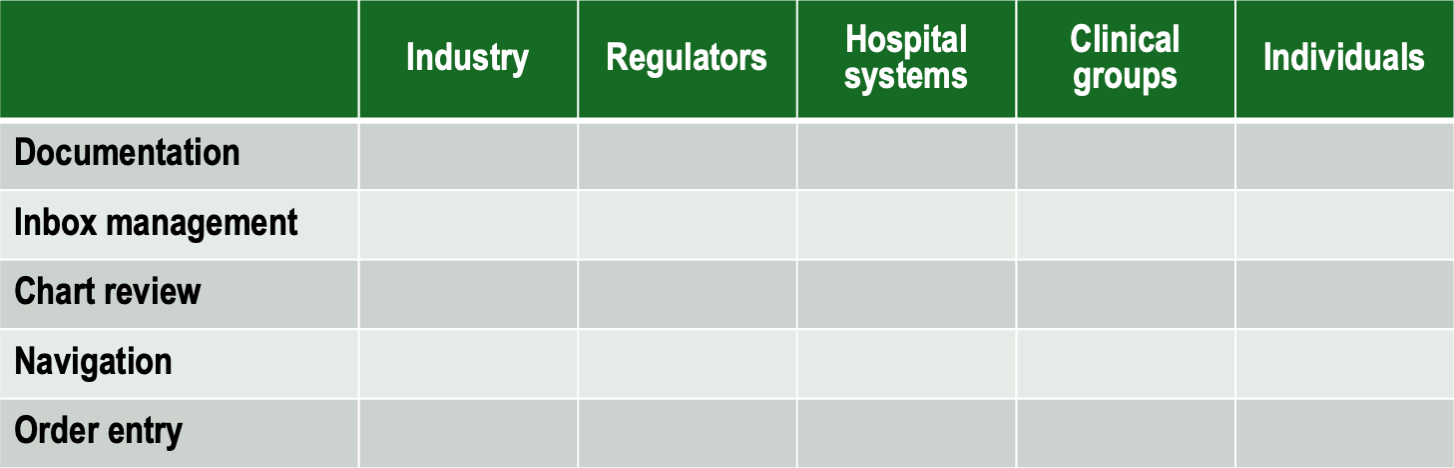
A proposed intervention matrix for considering improvements in the usability and intrusiveness of electronic health records.

Our study confirms (notwithstanding the limitations mentioned above) that the metrics of after-hours use are similar in range to those reported in other studies. When considering the JD-R framework, it may be helpful to consider as well the proposed matrix in Figure 2 as an approach to mitigating the intrusiveness of the EHR and optimizing system efficiency. The matrix itemizes the core activities for which clinicians use the EHR (ie, documentation, note management, chart review, navigation, and order entry) and considers the role played by industry, regulators, hospital systems, clinical groups, and individuals. System optimization from clinicians’ perspectives might include a range of interventions, such as improving the IT itself (eg, the structure of the user interface and system navigation, frequency and intrusiveness of alerts, and curation of information to limit information overload or chaos), streamlining regulatory requirements for using scribes or integrating artificial intelligence tools, adopting rules of engagement between system users (eg, when and how to communicate between members of interdisciplinary teams and roles and responsibilities pertaining to laboratory result reviews among team members), having hospital systems reappropriate clerical tasks to clerical support personnel to free up clinician time, and emphasizing personalization of the system for individual users (eg, templating, voice recognition software, and shortcuts). Interventions at all levels may be expected to rebalance after-hours time spent on the EHR. As per the JD-R model, increasing demands are not by themselves necessarily harmful as long as they are sufficiently supported by resources to accommodate them.

Ultimately, ours is an important longitudinal study on after-hours EHR use in the Canadian context with potential implications for clinician well-being and system usability.

## Supplementary material

10.2196/76872 Multimedia Appendix 1 Metrics included in the data analysis and their definitions, breakdown of participants by subspecialty, and timeline of observation periods.

## Abbreviations

EHR: electronic health record
FTE: full-time equivalent
JD-R: job demands–resources
REDCap: Research Electronic Data Capture
TOSH: time outside scheduled hours
