# Supplementary material for: After-Hours Use of the Electronic Health Record Among Medical and Surgical Specialists After Implementation of a System-Wide Integrated Clinical Information System in Alberta, Canada: Longitudinal Descriptive Study
Source: J Med Internet Res. 2026 Apr 15;28:e76872. doi: 10.2196/76872 (PMC13082444; doi:10.2196/76872)

**Supplemental Material**

**Supplemental Material Table S1.** Metrics included in the data analysis and their definitions

| **Metric** | **Value** | **Metric Calculation** |
| --- | --- | --- |
| Pajama Time | Average number of minutes a provider spent in charting activities on weekdays outside the hours of 7 AM to 5:30 PM or outside scheduled hours on weekends or non-scheduled holidays | *Numerator*: Minutes spent in charting activities outside 7 AM to 5:30 PM on weekdays and outside scheduled hours on weekends  *Denominator*: Number of scheduled days where time was spent in the system within the reporting period |
| Time outside scheduled hours | Average number of minutes a provider spent in the system outside of scheduled hours | *Numerator*: Minutes the provider spent in the system outside of scheduled hours based on Cadence (scheduling module) data with a 30-minute buffer before and the first appointment and after the last appointment  *Denominator*: Number of scheduled days where time was spent in the system within the reporting period |

**Supplemental Material Table S2.** Breakdown of participants by subspecialty

| **Medical subspecialty** | **Number of providers** | **Surgical subspecialty** | **Number of providers** |
| --- | --- | --- | --- |
| General internal medicine | 14 | General surgery | 9 |
| Nephrology | 10 | Orthopedic surgery | 3 |
| Neurology | 7 | Plastic surgery | 2 |
| Infectious diseases | 3 | Urology | 2 |
| Hematology | 3 | Cardiac surgery | 1 |
| Physical medicine & rehabilitation | 3 | Neurosurgery | 1 |
| Respirology | 3 |  |  |
| Cardiology | 2 |  |  |
| Geriatric medicine | 2 |  |  |
| Endocrinology | 2 |  |  |
| Gastroenterology | 2 |  |  |
| Dermatology | 1 |  |  |
| Intensive care | 1 |  |  |
| **Total** | **53** | **Total** | **18** |

**Supplemental Material Figure S1.** Timeline of observation periods


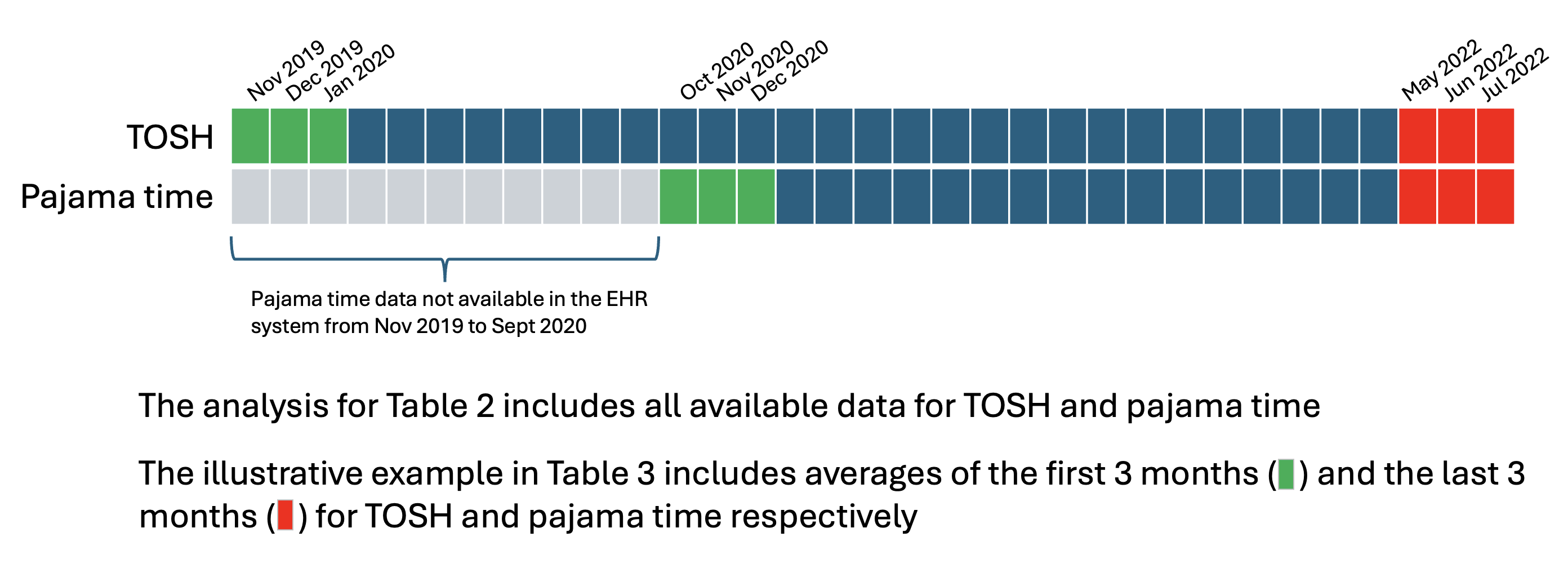

Supplement: Multimedia Appendix 1 [file jmir-v28-e76872-s001.docx]
